# A Distributed Architecture for Human-Drone Teaming: Timing Challenges and Interaction Opportunities

**DOI:** 10.3390/s19061379

**Published:** 2019-03-20

**Authors:** Karin Anna Hummel, Manuela Pollak, Johannes Krahofer

**Affiliations:** Department of Telecooperation, Johannes Kepler University, Altenberger Strasse 69, 4040 Linz, Austria; manuela.pollak@jku.at (M.P.); Johannes.Krahofer@gmx.at (J.K.)

**Keywords:** drones, UAVs (unmanned aerial vehicles), networked systems, network performance, human–robot interaction

## Abstract

Drones are expected to operate autonomously, yet they will also interact with humans to solve tasks together. To support civilian human-drone teams, we propose a distributed architecture where sophisticated operations such as image recognition, coordination with humans, and flight-control decisions are made, not on-board the drone, but remotely. The benefits of such an architecture are the increased computational power available for image recognition and the possibility to integrate interfaces for humans. On the downside, communication is necessary, resulting in the delayed reception of commands. In this article, we discuss the design considerations of the distributed approach, a sample implementation on a smartphone, and an application to the concrete use case of bookshelf inventory. Further, we report experimentally-derived first insights into messaging and command response delays with a custom drone connected through Wi-Fi.

## 1. Introduction

In recent years, employing mini-drones, also known as UAVs (unmanned aerial vehicles) and micro-aerial vehicles, in civilian applications has become popular. The capabilities of drones to screen an area quickly with on-board cameras and to establish wireless networks in the air [1,2] provide novel opportunities in search and rescue missions [3], 3D cartography, entertainment, (small) product delivery, smart farming, surveillance, and many other application domains. Yet, assuring the technical preconditions of safe and practical autonomous flight is challenging due to limited system reliability, short flight-time, and restricting legal regulations. Further, in realistic settings, autonomous drones will likely cooperate with humans, which is a new field of human–computer interaction.

Employing drones in human-drone teams involves the coordination of autonomous drone behavior and user interactions. A major challenge is to find a fitting architecture that supports both aspects. One unconventional option to follow is a distributed system design, where only basic control is implemented on the drone’s microcontroller and computationally-intense tasks of drone behavior are executed on a more powerful remote computer, or even in a cloud computing infrastructure. The on-board controller is basically used to receive commands and to execute them similar to manual remote control. Such a distributed architecture is advantageous as it overcomes limitations in applying, e.g., machine learning, image processing, and big data analysis on drones. Further, human interactions can be integrated well into the remote control loop. On the downside, communication delays occur in this distributed architecture.

To split drone applications between the physical drone and a remote computer, the drone has a model-based digital representation on the remote machine. The remote drone model is used to process the control logic based on sensory input such as camera images received from the physical drone. The outcomes of the control logic are commands sent back to the drone, which adjusts its behavior accordingly. Since control is a real-time task, there is a need for characterizing delays and their effect on task decisions, in particular when communicating through standard wireless communication networks.

Our main focus is on investigating whether a distributed architecture and distributed control loop to decide on the next actions are feasible and which delays have to be considered. Hereby, our assumptions are that the drone provides camera images as a primary sensor input and that a human is integrated in the system to form a team with the drone. To give first insights into the problem, we present the implementation of a distributed architecture for human-drone teaming applied to a sample task, as well as quantitative and qualitative experimental results. In detail, we make the following contributions in the remainder of the paper:

- In Section 2, we present a survey of the state-of-the-art in control and timing-related drone research and on human-drone teaming with a focus on human–drone interaction.
- In general, drone systems are rather novel robotic systems and require a wide exploration of potential application fields. This is even more the case for human-drone teaming. We contribute by designing and implementing the functions necessary to provide a drone system for bookshelf inventory, which may be used by the research community as a sample test application. In Section 3, we describe this use case.
- We introduce a distributed architecture for human-drone teams and model timing in this architecture. Further, we demonstrate the feasibility of such a distributed architecture with a prototype app implementation running on a smartphone, as detailed in Section 4.
- We investigate the delay and aspects of the timing behavior in a real testbed with a Parrot Mambo drone equipped with an FPV (first person view) camera, operating through Wi-Fi in Section 5. Further, we provide qualitative insights for the specific use case of bookshelf inventory.

## 2. Related Work

Preparing drones for complex, autonomous tasks that involve humans has been investigated from various perspectives in the literature. Besides providing tools for deployment and remote testing, such as the deployment tool provided for drone swarms in [4] and learning-based testing of spatio-temporal properties in [5], understanding the real-time behavior of a drone system is crucial for operation. Hereby, the control loop and the timing characteristics of autopilots are most demanding. Another challenge is to define appropriate interfaces for human–drone interaction.

### 2.1. Control and Timing

Controlling the flight behavior of modern micro-drones is supported by autonomously-operating pilots such as the Ardupilot, a widely-used autopilot (http://ardupilot.org/). Typically, the navigation path is determined remotely, e.g., by setting GPS waypoints in outdoor scenarios, but specific motion control is performed locally on the drone using a low-level control loop that determines pitch, roll, and yaw movement. The control loop is usually time-driven, i.e., periodically invoked with a constant rate. An event-based, reactive approach is suggested in [6,7]. By adapting the control rate dynamically based on environmental needs such as wind gusts and pressure gradients, a more efficient scheme can be derived that saves energy, leading to an extension of the flight-time. Besides, more accurate drone motion is achieved. In particular for indoor drones, vision is used for navigation, obstacle identification, and obstacle avoidance. In [8], it is shown that vision-based navigation works well even for very small drones termed pocket-drones that weigh only 40 g. In contrast to these local control approaches, our architecture allows distributing parts of the control loop between the drone and a remote computer.

Modeling the control loop allows understanding the drone’s behavior, to proof characteristics, and to perform exhaustive tests. A particular aspect to model is the timing behavior. As in any other cyber-physical system, the time between sensing, processing, and actuation has to remain below a constraining bound. Concerning drones, information at least from inertial sensors and distance sensors has to be continuously evaluated to control the flight behavior of the aircraft by activating motors and rotors. To analyze the end-to-end time in the control loop, a pipe model is suggested in [9]. The model allows for analyzing the reaction time, as well as the freshness of events, thus assuring bounded operation at an early stage of development. The approach is validated by a port of the Cleanflight flight controller firmware to Quest (Intel Aero board). In [10], the Ardupilot is modeled with the aim to investigate timing behavior. With the model, it can be shown that the autopilot does not always meet the timing requirements, which is a severe problem for fail-safe operation. These erroneous executions are then also shown in real tests. Our work confirms the importance of accurate and bounded timing, as well as unexpected delays in the autopilot of the Mambo drone.

An approach similar to ours is taken by the work presented in [11]. A representative of a drone (or a swarm of drones) is leveraged to control the actual drone in the field and to get feedback from this field drone. This drone is termed the shadow drone. Besides real-time operation, the shadow drone can also be used during implementation and testing. Latency and disruption are mentioned as major challenges in [11]. In the paper, prototype implementations for the Parrot Rolling Spider and AR Drone 2.0 are described, yet an evaluation is missing. In our work, we consider a model as a representation of the physical drone used at the remote site, instead of operating a shadow drone. Furthermore, we present the results of an experimental study of communication delays that occur between the physical drone and the control unit running on an external computer.

To leverage the processing power of high-performance computers, drone and cloud integration is the natural next step. Related work has shown that integrating with a cloud can indeed provide benefits to autonomous vehicles. In [12], remote resources such as terrestrial antennas and cloud infrastructures are added to a local unmanned vehicle team (here, underwater vehicles). Computationally-extensive tasks are executed in the cloud. Simulations demonstrate that including cloud resources allows for performance improvements. Yet, this architecture considers a loosely-coupled distributed system where real-time behavior is not an issue. Differently, the cloud is used to support vision-based navigation in [13]. This real-time task makes use of cloud-based recognition of objects in order to avoid collisions. The latencies of image frame delivery are in the range of 10 s of ms to about 1 s. These latencies are confirmed by our study, which further evaluates the command response time.

### 2.2. Human–Drone Interaction

In addition to the autopilot control loop, our work targets the integration of humans and drones through suitable interfaces. This includes questions about how drones perceive humans and human interactions, as well as how humans perceive drones. Drones may use cameras to interact with humans, e.g., to avoid collisions with humans by applying obstacle avoidance methods and to react to human gestures. Alternatively, drones may use messaging to receive user input through an arbitrary device such as a remote control device, smartphone, smart watch, etc. In turn, drones may signal their status to the human. In [14], drone gestures are used to signal acknowledgment. The basic gestures are waggle, nod, and changing orientation. The drones used for experimentation are the Air-Robot AR-100B, Parrot AR 2, and Parrot Bebop 2. Based on user studies, the work found that distances of about 2 m are best to perform drone gestures and that orientation towards the human attracts human attention very well. A simple way of interaction is to just attract the user to follow a drone, which even works for visually-impaired humans following the sound emitted by a guiding drone [15]. Our aim in this work extends the basic interaction options by adding the collaboration aspect of human-drone teams. This means the drone and the human should work together to fulfill a given task. The human has to be aware of the solving degree of the task and requires a means to help the drone to proceed.

## 3. Sample Task: Inventory of a Bookshelf

To exemplify a joint human-drone team effort, we select the task of bookshelf inventory. The drone’s task is to team-up with a human in order to survey the available books in an institution, such as a public library. Integrating drones in such a task may allow speeding up inventories and involving humans preferably only in non-standard, difficult, and unclear situations. Furthermore, as drones can move in three dimensions, they are well suited to perform inventories even at high altitudes.

**Figure 1:**
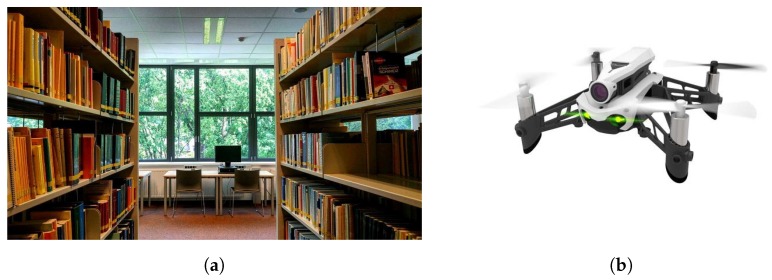
Bookshelf inventory components: (**a**) university library bookshelves and (**b**) Parrot Mambo equipped with a FPV (first person view) camera.

In detail, bookshelf inventory requires (i) counting the number of books available and (ii) detecting the book title, and potentially other information about the book. One option to simplify the task is to label the books with barcodes, QR-codes, or RFID (radio frequency identification) tags. Another option is to use image recognition to identify books and text recognition to derive the title, author, etc. The latter is advantageous as it does not require any particular tags on the books. Thus, we choose vision as the main input channel for performing the task and assume that the drone is equipped with an on-board camera. A sample library bookshelf of our university and our experimentation drone Parrot Mambo (https://www.parrot.com/eu/drones/parrot-mambo-fpv/) are depicted in Figure 1.

The drone operates indoors and mostly autonomously, i.e., it has to position itself close to the bookshelf at a starting position to perform the scanning task thereafter. As a starting point, the upper left corner of the shelf is searched for by finding the most left book in the top layer. Scanning is implemented as a traditional search behavior, i.e., one layer of the whole shelf is scanned after the other from the starting position following a zigzag movement pattern. The flight direction is alternated between flying from left to right and right to left. A major challenge is to move accurately along the optimal trajectory. The trajectory is calculated to allow the best placement for book recognition. Whenever a new image is provided by the drone’s camera, image recognition is invoked.

### 3.1. Image Recognition

Image recognition is aimed at identifying books, i.e., to find rectangles by evaluating the edge curvature of a contour and the number of edges (i.e., the angle has to be about 90${}^{\circ }$ and four edges need to be detected). Furthermore, the ratio between the book’s width *W* and height *H* (both in pixels) and the book surface *A* (in pixels) are evaluated. Finally, to differentiate between single books and a full shelf, a minimum amount of *n* books with a similar height of *H* pixels is required.

To recognize books in real libraries, books with different shapes have to be identified. Thus, a pre-phase has to be considered to classify and learn the different types of books available in one library. In our implementation of the bookshelf inventory, we make a few simplifying assumptions. First, we assume that the books are of similar size and that the height lies in the interval of $\left[H-100,H+100\right]$ pixels. Further, in our configuration, the following shape conditions have to be fulfilled: H≥3×W pixels, A≥1500 pixels, and n≥5 (this configuration has been experimentally derived and will be subject to change in other settings).

### 3.2. Autonomous Navigation

The drone should perform autonomous, vision-based navigation in order to solve the inventory task. The drone’s state machine is designed to solve the task by splitting the drone operation into the following states:

- Take-off: This is the initial state of the drone when it starts its flight.
- Find start position: After taking off, the drone needs to identify the top-left book by combining image recognition to detect books and re-positioning to get the best position for image recognition. In detail, the drone changes the distance to the bookshelf to identify the starting position and then navigates to this position until the top-left book is in the center of the view. Then, the drone changes to the scanning state.
- Scan: Depending on the layer, the drone scans books from left to right or right to left. The altitude is lowered when one layer is totally scanned.
- Finish: When no more shelf layers are left, the drone lands.

The drone uses various movement behaviors to assure that a stable position can be maintained as demanded by the states. Naturally, the outcome of the inventory task heavily depends on the methods used for image detection. We will detail the implementation of navigation and image recognition in Section 4.3.

## 4. Architecture Description and Implementation

Any drone application requires control of flight behavior in order to support the specific task of the drone. Traditionally, autonomous flight behavior is implemented on the drone in order to avoid delays and to access the auto-pilot directly. Yet, this architecture limits the computation capabilities; thus, we follow a novel direction in drone application design by proposing a distributed architecture.

### 4.1. Distributed Architecture

With a distributed design, we aim at overcoming the limitations of small-footprint embedded processors on drones. This comes with the advantage of speeding up algorithms that require substantial resources such as machine learning and image recognition. Our first design decision is to distribute the drone logic between the drone and a remote computer with more advanced computation capabilities, keeping only low-level piloting at the drone. A second design decision originates from our aim to support human-drone teams; thus, we require a mobile computer or interface for including the human in the team. Finally, the drone’s task is to support the remote logic with sensor-based input data, which have to be generated by the drone.

**Figure 2:**
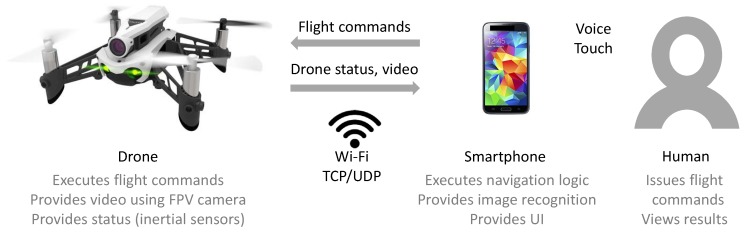
Distributed architecture for human-drone teaming in the concrete case of a camera-equipped drone and a smartphone app providing the user interface for the human.

Figure 2 visualizes a concrete case of such a distributed architecture, capable of supporting the use case bookshelf inventory described in Section 3. The drone captures and sends video data using the on-board FPV (first person view) camera, performs flight maneuvers, and sends status information about the current directional speed. A smartphone app receives the drone’s information, evaluates the image and status data of the drone, and sends back flight commands. In addition, the smartphone app provides voice recognition and a touch interface to allow the human team-mate to issue flight commands and displays the progress of the scanning task. The communication technology is Wi-Fi due to the large bandwidth required for video transmission; standard TCP/UDP messages are used to communicate with the drone. The drone provides the Wi-Fi network and acts as an access point and DHCP server. The smartphone connects to the Wi-Fi network as a client device. Image recognition is performed on the smartphone (note that the concept of the distributed architecture is not limited to smartphones, camera input, or to the sample use case).

The interaction process between the human and the drone is designed in a way that the human can interrupt the drone’s autonomous navigation and change to manual navigation at any point in time. Manual navigation is possible by voice and touch input. When voice input is turned-on, predefined voice commands (i.e., up/down, forward/backward, and left/right) are received through the smartphone’s microphone and interpreted in the app by leveraging Android’s voice recognition service. In addition to voice input, the human team-mate may employ the smartphone as a remote flight controller by pressing buttons of the smartphone app. By interacting with the drone, the human may support sophisticated navigation tasks, such as finding the bookshelf of interest in a room. Whereas this is an easy task for the human, it requires semantic knowledge about the room objects and advanced vision-based navigation by the drone.

### 4.2. Timing

**Figure 3:**
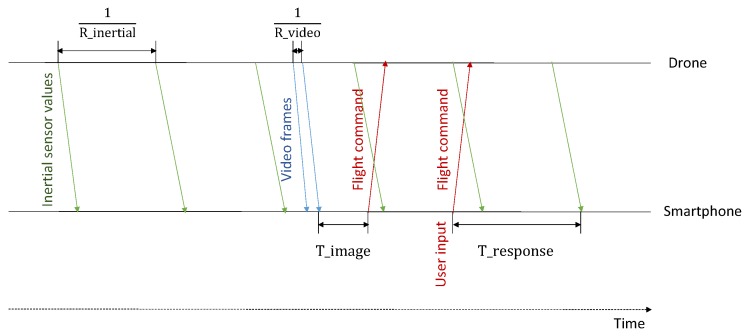
Times to consider in a vision-based distributed navigation system: Inertial sensor values are sent periodically from the drone to the smartphone, as well as video frames. After processing an image, the navigation logic decides upon the next flight maneuver and sends a flight command to the drone. At any point in time, the human user may interfere and send an overruling flight command to the drone via interacting with the smartphone.

A consequence of the distributed architecture is the more complex timing and message transfer needed, as depicted in Figure 3. The drone sends status information to the smartphone at a constant rate of R_inertial as well as a video stream at a rate of about R_video, which is an H264-specific video rate. Upon reception of a frame that is part of the video stream, the smartphone app analyzes the frame by performing image recognition and may send a flight command back to the drone when needed after a processing delay of T_image. Yet, flight commands can also be triggered by a human at any point in time. The time it takes for receiving information about the effect of the flight command is defined as T_response. The control loop has to consider image information, but also T_image and the expected T_response in order to count-in ongoing drone movement that happens in parallel with control decisions.

### 4.3. Implementation

In this section, we detail the hardware and software employed in the distributed system, how vision-based navigation control is implemented, and messaging between the remote app software and the drone.

#### 4.3.1. Hardware and Software

A developer drone is required that is capable of communicating wirelessly with any computer, providing basic piloting functionality for indoor flights upon reception of commands, and sending back status information and video streams for further processing. Among the possible drones, the Parrot Mambo provides the properties needed. Table 1 summarizes the basic hardware and software capabilities of the drone. This quadcopter is easy to operate, can be equipped with an FPV camera of sufficient resolution, and is light-weight. With the FPV camera, Wi-Fi is supported and can be leveraged for communication.

The user interface, image and text recognition, and autonomous trajectory calculation are performed by an app running on a smartphone. The smartphone provides sufficient processing power for vision-based navigation. The app further provides voice recognition and a touch-based interface, which allows the user to manually navigate the drone, e.g., when the drone is not able to find the correct position for scanning. User commands are executed with the highest priority, meaning they overrule autonomous navigation. The app is developed to run on any Bluetooth- and Wi-Fi-enabled smartphone running Android Versions 4.4–8.1. The app was successfully run on four different smartphone platforms, among them a Samsung Galaxy S5 (CPU quad-core with 2.5 GHz, 2 GB RAM, Bluetooth, and IEEE 802.11n/ac).

The app is a Java-based custom-developed Android app leveraging the Parrot developers’ classes available to connect from an Android application to the drone using TCP (https://github.com/Parrot-Developers/Samples/blob/master/Android/). The OpenCV library is used to detect books as rectangles (OpenCV library Release 3.4.1, http://opencv.org/). For text processing of book titles, in the current implementation, the external API Tesseract OCR for Android is employed (Tesseract library tess-two Version 5.4.1, https://github.com/rmtheis/tess-two/). Among possible other text recognition options, this stands out as it can provide recognition without connecting to a remote server. All results, as well as flight information are saved persistently on the smartphone’s memory.

#### 4.3.2. Image Recognition and Drone Navigation Control

The core parts of the application running on the smartphone are *image recognition* and *drone navigation control*. Image recognition takes the camera picture sent by the drone to identify books as rectangles. The output of the application is a scanning result consisting of the number of scanned books and an estimate of book titles. Beyond counting books, image recognition is at the same time leveraged for drone navigation control.

The remote navigation algorithm implements the state model introduced in Section 3 as follows: (i) first, the drone is positioned at the start position for scanning; (ii) then, it is commanded to scan a bookshelf layer by layer; and (iii) it is commanded to land when finished. To determine the drone’s position and to give a command for the next drone movement, the algorithm takes the camera images sent by the drone as input, leverages image detection to detect rectangles, analyzes the position of the rectangles on the image area, and bases the next movement on the result of image and geometry analysis.

The process of image recognition runs in parallel to the other drone activities. Obstacles that have the contour of a rectangle with a book shape are detected as books characterized by four vertices, 90 degree angles, two opposite sides of equal length, and fulfilling a configurable constraint concerning the relation between the length and the width of the rectangle, see Section 3 for the specific configuration. The outcome of image processing is not only important for counting books, but also provides input for drone navigation decisions that are vision-based. Remote drone navigation control performs the following steps depending on the drone’s state:

- Take-off: In this state, the drone is commanded to move up until a configurable height is reached. In the current implementation, it is assumed that the drone is positioned manually by the user so that the orientation of the drone is towards the bookshelf of interest. Note that correcting the orientation of the drone is a typical example of human intervention, which allows avoiding time-consuming autonomous search for the correct bookshelf in the hall.
- Find start position: The aim is to detect the top-left book and to stabilize when the book is in the center of the camera view. The movement of the drone is controlled accordingly. First, the drone is commanded to increase the distance to the shelf in order to detect the top-left book (no other books scanned higher, no other books scanned more left), then it moves up and left to stabilize with the top-left book at the center of the image (blurring of 10% is calculated in). If the shelf is too high, the human user may again help and navigate the drone to a position where the upper left corner of the shelf can be detected in the image. To reach this stable position, both back and forward and right and left movements are performed autonomously. Due to moving too far to the left or right (or up or down), it may take some time to stabilize. This state is left to the scanning state when the top-left book is in the center of the camera image.
- Scan: In this state, the drone is commanded to scan the books in one layer from left to right and in the next layer from right to left, and so on. The altitude is lowered when one layer is totally scanned, which requires again a positioning of the drone to center on the first book of the new layer. After this movement is finished and the drone is stable, it starts scanning the books in the particular layer and counts all books found. During scanning, the drone aims to keep a distance that is optimal for scanning the book titles as well. The distance is kept by evaluating the ratio of the book size to the overall picture size. If the book is too large, the distance is too small, and the drone has to zoom out by flying backwards, and vice versa. The current configuration settings aim for a book size of 20–30% of the picture size required for a stable distance.
- Finish: This is the end state of the algorithm. When no layer is left, the drone is commanded to land.

To achieve the desired drone behavior, the remote navigation control algorithm sends flight control messages to the drone, which, after a transmission delay, are executed by the drone.

#### 4.3.3. Flight Control Messages

Flight control messages are used to interface between the smartphone app and the drone. These high-level control messages are based on the Parrot messaging API. Hereby, sending messages is either forced by the navigation control algorithm or by the human through the app. A flight command consists of a tuple of parameters describing the speed and angle of movement in 3D along the traditional axis of an aircraft: pitch, yaw, and roll. The parameters are packed into a flight command and sent via a TCP message to the copter. The copter will perform a 3D movement according to the flight command (i.e., moving up/down, forward/backward, or left/right).

On the smartphone, command processing includes autonomous generation of the flight commands based on the status of the drone, its current position, and a calculation of the required movement to reach the next position as determined by the task. In addition, flight commands are generated when the user takes over control and navigates the drone. On the drone, safe and reliable flight command processing is provided by Parrot’s on-board control logic, which is responsible for controlling the rotors to achieve the movement specified by the flight command. In return, the actual flight characteristics of the copter are sent back to the smartphone app using the same set of parameters. The parameters are:

- Gaz: With this parameter, the vertical speed is adjusted, leading to a change of throttle (rotor movement) on the copter and an upward (downward) movement. It is expressed as a percentage of the maximum vertical speed.
- Pitch: This parameter describes the angle towards the sky/ground, expressed as a percentage of the maximum angle. By adjusting the pitch, forward (pitch towards the ground) or backward (pitch towards the sky) movement is achieved.
- Roll: The roll angle expresses the tilt of the copter, again expressed as a percentage of the maximum possible tilt of the copter. The roll angle controls side movements of the copter, i.e., moving to the right or to the left.
- Yaw: This parameter controls the heading and is expressed as a percentage of the maximum yaw rotation speed.

## 5. Experiments and Results

We are mainly interested in investigating the timing behavior and book detection quality. To discuss these characteristics, we conducted experiments with our testbed consisting of one drone and one smartphone and present the collected measurement results.

### 5.1. Experiment Setup

All experiments are executed with the off-the-shelf drone Parrot Mambo. The drone provides the Wi-Fi network, while the human team-mate connects to the drone using an Android smartphone. Basic latency tests are performed with a custom ping utility running on the smartphone, while the task-specific experiments are performed using the inventory scanning app. During the task-specific tests, the drone also streams video to the smartphone.

Three different experiments are conducted to investigate the following characteristics:

- Latency and reliability: With the ping utility program, ICMP (Internet Control Message Protocol) packets are sent to the drone over a period of five minutes. This experiment aims to investigate the basic latency and reliability of the Wi-Fi connection.
- Command response time: The drone is commanded to go up with a speed of 20% and then down with the same speed of 20% (meaning 20% of the maximum drone speed of up to 30 km/h). This experiment is repeated ten times. Overall, 20 flight command messages are sent setting the up (down) speed to gaz=20 (gaz=−20); see Section 4.3 for details of the flight command messages. For each of the 20 commands sent, the response time is measured. Each command is executed for two seconds. As the drone sends status information back with a rate of 1/500 ms${}^{-1}$, we investigate the effect in two command frequency settings: (i) same command frequency: here, a new command is sent every two seconds; we expect a synchronization effect due to the fixed command response frequency; and (ii) random command frequency: in this setting, sending of commands is triggered with a random rate, where the inter-command time is equally distributed in the interval of $\left[2,2.5\right]$ s.
- Quality of recognition: This test is performed to give first insights about book recognition performance in the distributed architecture and the effect of positioning. In the experiment, a poster is leveraged to mimic a bookshelf. This is a simplification of a real library setting and thus exhibits limitations, yet it is advantageous for reasons of reproducibility. The poster contains three layers of books; book spines are of different colors; and titles vary in font size. We exemplify the effect of distance between the drone and the book shelf and discuss these insights qualitatively. We examine three different distances: 0.5 m, 1 m, and 1.5 m. The number of correctly identified books is measured.

### 5.2. Latency, Reliability, and Command Response Time

**Figure 4:**
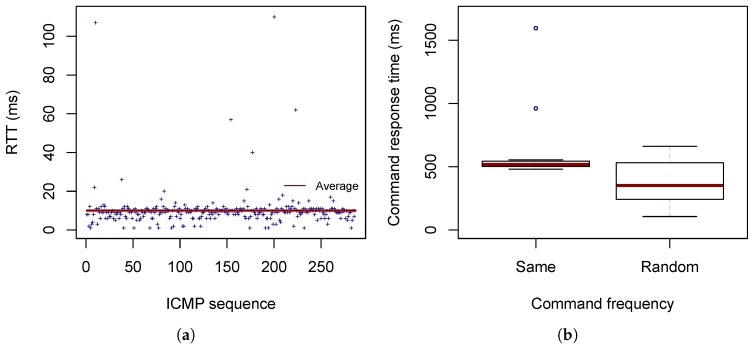
Investigation of delays: (**a**) RTT (round trip time) recorded over a period of five minutes (286 ICMP messages) and (**b**) command response time with a constant inter-command time of 2 s (same frequency) and an inter-command time randomly distributed in the interval of [2,2.5] s (random frequency). The red middle line in the box plots corresponds to the median, and the upper and lower borders of the box visualize the 25% and the 75% quartiles, respectively.

The latency is measured in terms of RTT (round trip time). The smartphone is placed 1.5 m away from the drone, connected to the drone Wi-Fi network, and sends ICMP messages of a size of 64 bytes to the drone. The experiment lasts about five minutes; 286 messages are transmitted. The results show a stable Wi-Fi connection with 0% loss. The average RTT is 10 ms (minimum: 1 ms, maximum: 110 ms, standard deviation: 10.1 ms). Figure 4a depicts the measured RTT values and the average.

The measured response times show that with the default setting of sending drone status information every 500 ms and sending commands to the drone with a random rate the mean delay is 376.1 ms (594.1 ms in the case of a constant command rate). It is worth mentioning that there were rare cases in which the command took a few response cycles until the expected confirmation was received with a delay of up to 1595 ms (constant command rate), which may lead to over-reactions in the automatic control loop. Figure 4b and Table 2 detail the measurement results.

### 5.3. Inventory Task: Qualitative Discussion

**Figure 5:**
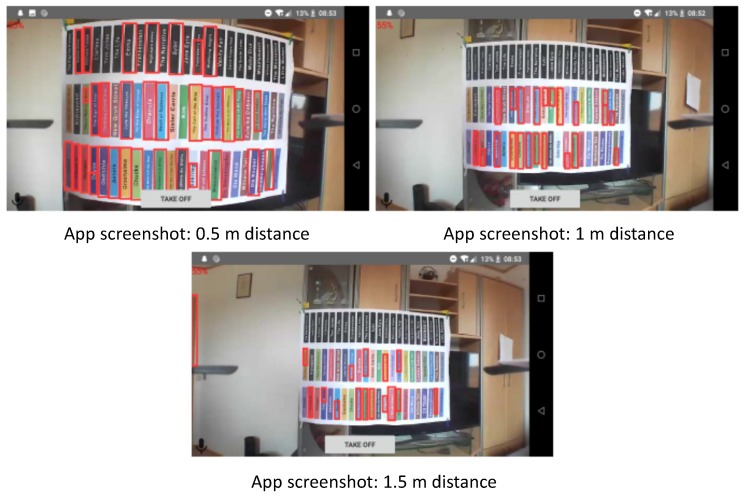
Visualization of book detection results marked as red rectangles, observed at three different distances.

While the basic functionality of book and text recognition is working, the success rate of identifying books heavily depends on the capabilities of image processing, which is currently the limiting factor. As visualized by Figure 5, only a fraction of the books on the poster were detected, even when positioned at the best spot . The success rate does not improve by moving along the optimal trajectory for scanning; thus, the navigation algorithm is not the cause of the low success rate. (Note that in the screenshots, the whole bookshelf fits in one image frame for reasons of visualization. This is not required by the detection algorithm.)

The experiments indicate that distance has a high impact on the number of books that can be detected. Table 3 summarizes the results of one experiment, i.e., the amount of books found at each distance and the decrease of success related to the best scan result at 0.5 m. A significant decrease to 0.64 (0.45) at a distance of 1 m (1.5 m) demonstrates the impact of distance on detection success (the results of text processing are not discussed here, as they were not convincing due to the limitation of the text recognition library).

## 6. Conclusions

This paper presents a distributed architecture for human-drone teams in which the computationally-intensive task of image recognition is off-loaded from the drone to a smartphone. The smartphone further provides an interactive app with speech recognition and a touch interface for human–drone interaction. Flight-control decisions are not taken on-board the drone, but on the smartphone where images recorded by the drone’s camera are processed to determine next flight maneuvers. We demonstrated the feasibility of the concept by a prototypical implementation of the architecture applied to the sample task of book inventory. Our first experiments reveal that the average network latency is as low as expected from a direct aerial Wi-Fi link (10 ms), and the command response time is a few 100 s of ms on average. Yet, we experienced artifacts with a command response time of above 1.5 s, which may be critical to perform accurate positioning. With the current implementation, image recognition is the limiting factor. Yet we could demonstrate that the distance to the bookshelf has a notable impact on book recognition. In our future work, we will extend the experiment-based study of the timing behavior of the distributed architecture and will also work on improving the limits identified concerning image recognition.

## Figures and Tables

**Table 1:** Overview of the testbed drone hardware and software.

	Size, Weight	CPU	Sensors	Lifetime	Software
Quadcopter	180×180×40 mm,	800 MHz	pressure, gyroscope	10 min,	firmware v3.0.17
Parrot Mambo	70 g (with battery)	ARM	3-axis accelerometer,	660 mAh	
		processor	vertical camera		

**Table 2:** Overview of command response time results.

Experiment	Median	Mean	Variance	Min	Max
Same frequency	518.5 ms	594.1 ms	65.7 ms	481 ms	1595 ms
Random frequency	351.5 ms	376.1 ms	29.3 ms	106 ms	661 ms

**Table 3:** Number of books found in the experiment poster shelf containing 66 books at different distances (including the detection rate). The detection rate in the last column is the number of books found related to the number of books detected in the best case of our experiments, i.e., 33 books at a distance of 0.5 m.

Distance	Number of Books Found	Detection Rate Related to Best
0.5 m	33 (0.5)	1 (best)
1 m	21 (0.32)	0.64
1.5 m	15 (0.22)	0.45
